# A plastidic starch biosynthetic pathway defines columella-specific carbon metabolism in rice root tips

**DOI:** 10.3389/fpls.2026.1852363

**Published:** 2026-06-01

**Authors:** Seok-Hyun Choi, Jin-Hyeong Lee, Md Mizanor Rahman, Sang-Kyu Lee, Jong-Seong Jeon

**Affiliations:** 1 Graduate School of Green-Bio Science & Crop Biotech Institute, Kyung Hee University, Yongin, Republic of Korea; 2 Division of Life Science, Plant Molecular Biology and Biotechnology Research Center, Gyeongsang National University, Jinju, Republic of Korea

**Keywords:** AGPase, OsGPT1, plastidic metabolism, rice, root-tip columella, starch biosynthesis

## Abstract

Starch biosynthesis in rice (*Oryza sativa*) is precisely regulated in an organ-specific manner, but the molecular mechanism operating this pathway in root-tip columella cells has remained unclear. Here, we elucidated the starch biosynthetic pathway in rice root tips using CRISPR/Cas9-mediated mutagenesis, Lugol’s iodine staining, expression analysis, anatomical validation, and quantitative analysis of starch and soluble sugar contents. Systematic genetic screening revealed that disrupting genes encoding individual large (AGPL) or small (AGPS) subunits of ADP-glucose pyrophosphorylase (AGPase) did not affect columella starch accumulation, indicating functional redundancy. Expression profiling and double-knockout analysis demonstrated that starch biosynthesis in columella amyloplasts specifically requires the plastidic AGPase subunits OsAGPL1, OsAGPL4, OsAGPS1, and OsAGPS2a, whereas cytosolic AGPase subunits are dispensable for this pathway. Subunit-specific disruption further showed that OsAGPS2a, but not OsAGPS2b, is essential for starch formation in columella cells, establishing the exclusive requirement for plastidic AGPase function in this tissue. We also show that glucose-6-phosphate (Glc-6-P) import into columella amyloplasts is mediated by Glc-6-P/Phosphate Translocator 1 (OsGPT1), whereas OsGPT2 paralogs do not appear to contribute to this process. Loss of OsGPT1 function markedly diminished, but did not completely abolish, starch accumulation. These changes were accompanied by lower soluble sugar levels, suggesting that disrupting plastidic starch biosynthesis impairs sink strength in root-tip tissues. Analysis of resin-embedded sections confirmed that starch deposition is restricted to columella amyloplasts and is dependent on plastidic AGPase and OsGPT1 function. Together, our results define a complete plastidic starch biosynthetic pathway in rice root-tip columella cells and establish this tissue as a distinct, locally specialized metabolic sink within the organ-specific carbon partitioning network of rice.

## 1 Introduction

Starch accumulation in plastids is a fundamental metabolic feature that supports diverse physiological functions in plants. In rice (*Oryza sativa* L.), starch is synthesized in the grain endosperm and in multiple vegetative and reproductive tissues, including leaves, stems, pollen grains, and root tips. Although the physiological significance of starch differs among these organs, accumulating evidence indicates that individual tissues employ specialized starch biosynthetic routes that reflect their distinct metabolic roles and carbon flux demands (Ohdan et al., 2005; Lee et al., 2007; Okamura et al., 2013; Lee et al., 2022).

Starch biosynthesis requires a coordinated sequence of enzymatic reactions in plastids. Glucose-6-phosphate (Glc-6-P) is converted to glucose-1-phosphate (Glc-1-P) by phosphoglucomutase, and ADP-glucose pyrophosphorylase (AGPase) catalyzes the formation of ADP-glucose (ADP-Glc), the immediate glucosyl donor for starch polymerization. AGPase is a heterotetramer (L_2_S_2_) composed of two large (AGPL) and two small (AGPS) subunits; AGPase activity is only observed in the heterotetramer (Ballicora et al., 2003; Georgelis et al., 2009). Previous studies have established that distinct subunits of rice AGPase differ in their predicted and experimentally supported subcellular localizations. In particular, OsAGPL2 and OsAGPS2b lack plastid-targeting signals and localize to the cytosol, whereas other AGPLs and AGPSs contain N-terminal transit peptides and are targeted to plastids (Lee et al., 2007). Notably, *OsAGPS2* is a single genetic locus that generates two different proteins through alternative transcription initiation, producing plastid-localized OsAGPS2a and cytosolic OsAGPS2b (Lee et al., 2007). Although the biochemical reaction catalyzed by AGPase is conserved, the subcellular routing of carbon toward starch biosynthesis differs markedly among tissues, particularly in cereals.

In photosynthetic leaves, starch biosynthesis occurs exclusively in chloroplasts via plastid-localized AGPase, predominantly involving the subunits OsAGPS2a and OsAGPL3, and primarily functions as a transient diurnal carbon reserve (Lee et al., 2007). By contrast, the developing endosperm of cereal grains mainly relies on cytosolic AGPase, primarily composed of OsAGPS2b and OsAGPL2, to generate ADP-Glc in the cytosol, which is subsequently transported into amyloplasts to support massive starch accumulation during grain filling under hypoxic conditions (Lee et al., 2007; Lee and Jeon, 2020).

Beyond these well-characterized tissues, starch also accumulates in vegetative sink organs such as the stems of rice plants, where it serves as a temporary carbon store that is later remobilized to support grain filling. Genetic analysis has revealed that starch biosynthesis in the rice stem is controlled by a distinct set of AGPase subunits. A mutant lacking plastid-localized OsAGPL1 exhibits an almost complete loss of starch in its stems while showing no major defects in overall plant growth or grain yield, indicating that starch biosynthesis in stems is highly dependent on plastidic AGPase activity and operates largely independently of starch metabolism in endosperm (Cook et al., 2012; Okamura et al., 2013). This finding provided early genetic evidence that rice deploys organ-specific AGPases to establish discrete starch storage pools, highlighting the plasticity of carbon partitioning among tissues.

Subsequent studies have extended this concept of tissue-specific metabolic specialization to other organs. In rice pollen grains, starch biosynthesis depends on hexose phosphate metabolism in plastids rather than cytosolic ADP-Glc production, with genetic evidence demonstrating the requirement for the plastid-localized subunit OsAGPL4; the associated small AGPase subunit remains unresolved. These findings support the notion that heterotrophic tissues can adopt plastid-localized starch biosynthetic routes that differ fundamentally from those operating in the endosperm (Lee et al., 2016). Collectively, these findings indicate that starch biosynthesis in rice follows a modular organizational principle, in which different tissues recruit distinct combinations of AGPase large and small subunits and carbon import pathways. In plastid-localized starch biosynthetic routes, carbon entry into plastids is mediated by hexose phosphate transporters, particularly Glc-6-P/Phosphate (Pi) Translocator (GPT), which imports Glc-6-P across the plastid envelope to supply the necessary substrate for plastidic AGPase activity (Lee et al., 2016; Zhang et al., 2021).

Despite these advances, the starch biosynthetic pathway operating in columella cells in the rice root tip, a region that preferentially accumulates starch in amyloplasts, remains undefined. The root tip represents a dynamic sink tissue that continuously imports and utilizes photoassimilates to sustain root growth and development. Because root system performance directly influences water and nutrient acquisition, sink activity in root tissues is closely linked to whole-plant productivity. Thus, starch biosynthesis in root-tip cells is likely to play a role in local metabolism and in shaping carbon partitioning across the plant. In this context, two mechanistically distinct routes are theoretically possible: cytosolic AGPase could generate ADP-Glc in the cytosol for subsequent import into amyloplasts, analogous to the endosperm pathway; or Glc-6-P could be imported into amyloplasts, where plastid-localized AGPase locally produces ADP-Glc. In Arabidopsis (*Arabidopsis thaliana*), starch biosynthesis in columella cells follows the latter route and depends on plastidic AGPase together with the transporter GPT1 (Niewiadomski et al., 2005; Tsai et al., 2009), but whether a similar mechanism operates in the root-tip cells of the monocotyledonous species rice has not been established. In addition, the specific subunit compositions and carbon import routes of AGPase operating in columella cells in rice root tips have not been defined.

In this study, we systematically dissected the starch biosynthetic pathway in rice root-tip columella cells using clustered regularly interspaced short palindromic repeats (CRISPR)/CRISPR-associated nuclease 9 (Cas9)-mediated mutagenesis, Lugol’s iodine staining, gene expression analysis, anatomical validation, and quantitative analysis of starch and soluble sugar contents. We demonstrate that starch accumulation in columella amyloplasts requires the plastidic AGPase subunits OsAGPL1, OsAGPL4, OsAGPS1, and OsAGPS2a, whereas cytosolic AGPase activity is dispensable. Furthermore, we identify OsGPT1 as the primary Glc-6-P transporter supplying the substrate for columella starch biosynthesis. Together, our findings establish a tissue-resolved, organ-specific framework for starch biosynthesis in rice and define root-tip columella cells as a distinct plastidic starch biosynthetic system within the broader network of organ-specific carbon allocation.

## 2 Material and methods

### 2.1 Plant materials and growth conditions

Wild-type rice plants (*Oryza sativa* L. ‘Dongjin’) and CRISPR/Cas9-generated mutant lines were used in this study. Rice plants were grown in a controlled growth chamber under long-day conditions (14-h light/10-h dark photoperiod, 25 °C day/20 °C night). Leaves and root tips were harvested from 6-week-old plants grown in the growth chamber for reverse-transcription quantitative PCR (RT-qPCR), Lugol’s iodine staining, and measurement of metabolite contents.

### 2.2 Generation of CRISPR/Cas9-mediated knockout plants

CRISPR/Cas9-mediated knockout lines were generated as previously described with minor modifications. Briefly, 20-nucleotide target sequences in the *OsAGPS*, *OsAGPL*, and *OsGPT* genes were selected using the CRISPRdirect web tool (https://crispr.dbcls.jp/) (**Supplementary Table 1**). Selected target sequences were synthesized, cloned into the pOs-sgRNA entry vector, and recombined into the pH-Ubi-Cas9–7 destination vector using the Gateway cloning system (Miao et al., 2013). For multiplex gene editing, CRISPR/Cas9 constructs were generated using the pRGEB32 vector based on the polycistronic tRNA–sgRNA strategy described previously (Xie et al., 2015). Briefly, two target sequences were assembled into a single dual-guide tRNA–gRNA cassette by PCR and Golden Gate cloning.

The resulting constructs were verified by colony PCR and Sanger sequencing before being introduced individually into Agrobacterium (*Agrobacterium tumefaciens*) strain LBA4404. Hygromycin resistance was used as a selection marker in plants. Rice transformation was performed via Agrobacterium-mediated transformation of seed-derived calli (Hiei and Komari, 2008). For single-gene knockouts, calli were transformed with individual constructs targeting *OsAGPL1*, *OsAGPL2*, *OsAGPL3*, *OsAGPL4*, *OsAGPS1*, *OsAGPS2*, or *OsGPT1*. For multiple-gene knockouts, CRISPR/Cas9 constructs carrying multiple target sequences were introduced to simultaneously target *OsAGPL1* and *OsAGPL4*, *OsAGPS1* and *OsAGPS2*, *OsAGPS1* and *OsAGPS2a*, or *OsAGPS1* and *OsAGPS2b*. For *OsGPT2* paralogs, a single sgRNA targeting a conserved region was used to simultaneously disrupt *OsGPT2-1, OsGPT2-2*, and *OsGPT2-3* (Xie et al., 2015).

Putative transgenic plants were screened by Sanger sequencing of the target sites to confirm insertion–deletion (InDel) mutations, and lines carrying homozygous or biallelic mutations were selected for all experiments. Due to severe sterility in some AGPase-deficient mutant lines, all plants, including wild type (WT) and transgenic plants, were maintained by ratooning and used for subsequent analysis to ensure consistent experimental conditions. Potential off-target candidates of the sgRNAs were predicted using Cas-OFFinder (http://www.rgenome.net/cas-offinder/).

### 2.3 Lugol’s iodine staining of starch granules

Lugol’s iodine staining was performed to visualize starch accumulation in rice root tips (Song et al., 2021). Newly emerging ratoon shoots were separated from the mother plants and grown independently for 6 weeks under the same conditions before root tip collection. Roots were fixed in FAA solution (5% formaldehyde, 5% acetic acid, 45% ethanol, v/v/v) for 24 h at 4 °C. Fixed samples were washed once with 50% (v/v) ethanol and stained with a 5% (v/v) diluted iodine–potassium iodide (I_2_–KI) solution (Sigma-Aldrich, St. Louis, MO, USA) for 2 min. After staining, the roots were briefly cleared using a trichloroacetic acid–phenol–lactic acid solution (2:1:1, v/v/v) for 3 min and observed under an Olympus SZX16 stereomicroscope (Olympus, Tokyo, Japan). At least three independent root tips were examined per genotype. Plants whose root tips showed an absence of (or less-intense) iodine staining were selected for analysis.

### 2.4 RNA isolation and RT-qPCR

Total RNA was extracted from rice tissues using RNAiso Plus (TaKaRa, Shiga, Japan) according to the manufacturer’s instructions. First-strand cDNA was synthesized from 0.5 μg of total RNA using ReverTra Ace qPCR RT Master Mix (TOYOBO) in a total reaction volume of 10 μL. The resulting cDNA was diluted five-fold in nuclease-free water prior to RT-qPCR.

Quantitative PCR was performed on a CFX Connect Real-Time PCR System (Bio-Rad, Hercules, CA, USA) with SYBR Green–based detection using GenetBio Prime Q-Master Mix (2×) (GenetBio, Daejeon, Korea) following the manufacturer’s instructions. Each reaction contained 3 μL of diluted cDNA as a template. Gene expression levels were normalized to *OsUBQ5* (LOC_Os01g22490), which served as an internal reference gene (Jain et al., 2006). Relative expression levels were determined using the 2^−ΔCt.^ method, where ΔCt was calculated as the difference between the Ct value of the target gene and *OsUBQ5*.

Primer sequences used for RT-qPCR of *OsAGPL1*, *OsAGPL2*, *OsAGPL3*, *OsAGPL4*, *OsAGPS1*, *OsAGPS2a*, *OsAGPS2b*, *OsGPT1*, *OsGPT2*, and *OsUBQ5* are listed in **Supplementary Table 2**. Primers for *OsGPT* family members were designed based on previously published criteria (Toyota et al., 2006).

### 2.5 Resin embedding and sectioning

For anatomical analysis, root tips were fixed in FAA solution for 24 h at 4 °C. Fixed samples were incubated overnight in phosphate-buffered saline (PBS, pH 7.4) containing 6.8% (w/v) sucrose and dehydrated through a graded ethanol series (30%, 50%, 70%, and 100% ethanol). The samples were then embedded in Technovit 7100 or Technovit 8100 resin (Kulzer, Frankfurt, Germany) according to the manufacturer’s instructions.

Embedded tissues were sectioned at a thickness of 10 μm using a rotary microtome (RM2165; Leica, Wetzlar, Germany). The sections were stained with a 10% (v/v) diluted iodine–potassium iodide (I_2_–KI) solution for 1 min, rinsed briefly with distilled water, and examined under an EX31 light microscope (Sunny Instruments, Yuyao, China).

### 2.6 Measurement of soluble sugar and starch contents

Approximately 20 mg of pooled root tips (fresh weight; FW) were harvested at Zeitgeber time 5 (ZT5) from newly generated roots of plants grown in a growth chamber. All samples were collected at the same Zeitgeber time to minimize variation in starch and soluble sugar metabolism caused by diurnal changes. Sucrose, glucose, fructose, and starch contents were quantified using a modified NAD(P)H-coupled enzymatic method as previously described, with slight modifications (Lee et al., 2005). Briefly, metabolites were extracted in 3.5% (w/v) perchloric acid and separated into soluble and residual fractions to measure soluble sugar and starch contents, respectively. Metabolite contents were normalized to root FW.

## 3 Results

### 3.1 Screening for knockout lines using a Lugol iodine staining assay reveals the redundant roles of individual AGPase genes in starch accumulation in root tips

**Figure 1:**
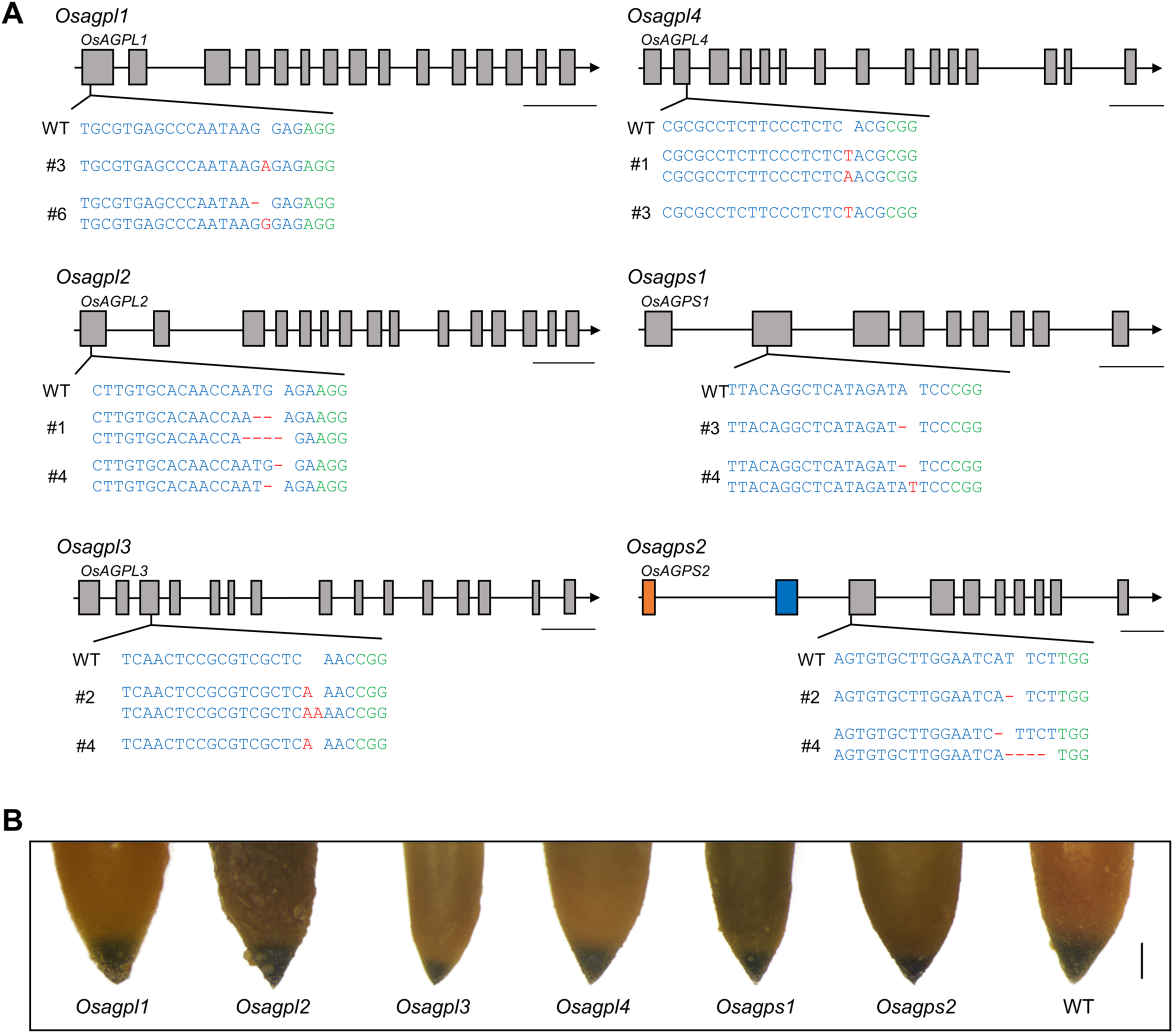
Lugol staining–based screening of AGPase isoforms involved in starch biosynthesis in root tips. **(A)** Schematic representation of CRISPR/Cas9 target sites and representative mutation profiles for each AGPase gene. Target sequences (blue), protospacer-adjacent motifs (PAMs; green), and indel positions (red) are indicated. Exons are represented as boxes and introns as lines. For *OsAGPS2*, which produces two distinct transcripts, exon 1a and exon 1b are indicated by blue and orange boxes, respectively. Scale bars = 0.5 kb. **(B)** Representative images of Lugol’s iodine staining of root tips from wild-type (WT) plants and single knockout lines for each AGPase gene. Scale bar = 200 µm. Root-tip images were digitally extracted and arranged on a white background for clarity.

To identify AGPase subunits required for starch biosynthesis in root-tip columella cells in rice, we established a Lugol’s iodine-based screening assay. We introduced CRISPR/Cas9 constructs targeting individual *AGPL* or *AGPS* genes (*OsAGPL1*, *OsAGPL2*, *OsAGPL3*, *OsAGPL4*, *OsAGPS1*, and *OsAGPS2*) into rice calli using Agrobacterium-mediated transformation. We selected transgenic rice seedlings carrying homozygous or biallelic mutations via Sanger sequencing of the target sites in each plant (**Figure 1A**). All analyzed mutant alleles were predicted to generate frameshift-induced premature stop codons (**Supplementary Figure 1**). The mutant seedlings were then stained at their root tips with Lugol’s solution to assess starch accumulation in columella amyloplasts (**Figure 1B**). In the root tips of WT (cultivar Dongjin) plants, columella amyloplasts accumulated starch and exhibited a characteristic dark-brown signal upon iodine staining.

We designed this assay to identify knockout lines lacking Lugol staining, which would indicate a defect in starch accumulation and thereby reveal which AGPase subunit(s) are essential for starch biosynthesis in root tips. Conversely, retention of normal staining would indicate that loss of a single AGPase subunit is insufficient to disrupt starch formation. All single-knockout lines, including those targeting AGPase subunits whose expression patterns and subcellular localizations were previously shown to differ (Lee et al., 2007), retained Lugol staining in root-tip columella cells (**Figure 1B**). Quantification of the Lugol-positive region length revealed no statistically significant differences between WT and the analyzed single mutants, although *Osagpl3* showed a relatively lower mean value (**Supplementary Figure 2**). These results suggest that starch biosynthesis in root-tip columella cells is maintained through functional redundancy among AGPase subunits, as disruption of any single gene did not abolish Lugol-positive starch accumulation.

### 3.2 Redundant pairs of plastid-localized AGPase subunits are required for starch biosynthesis in root tips

**Figure 2:**
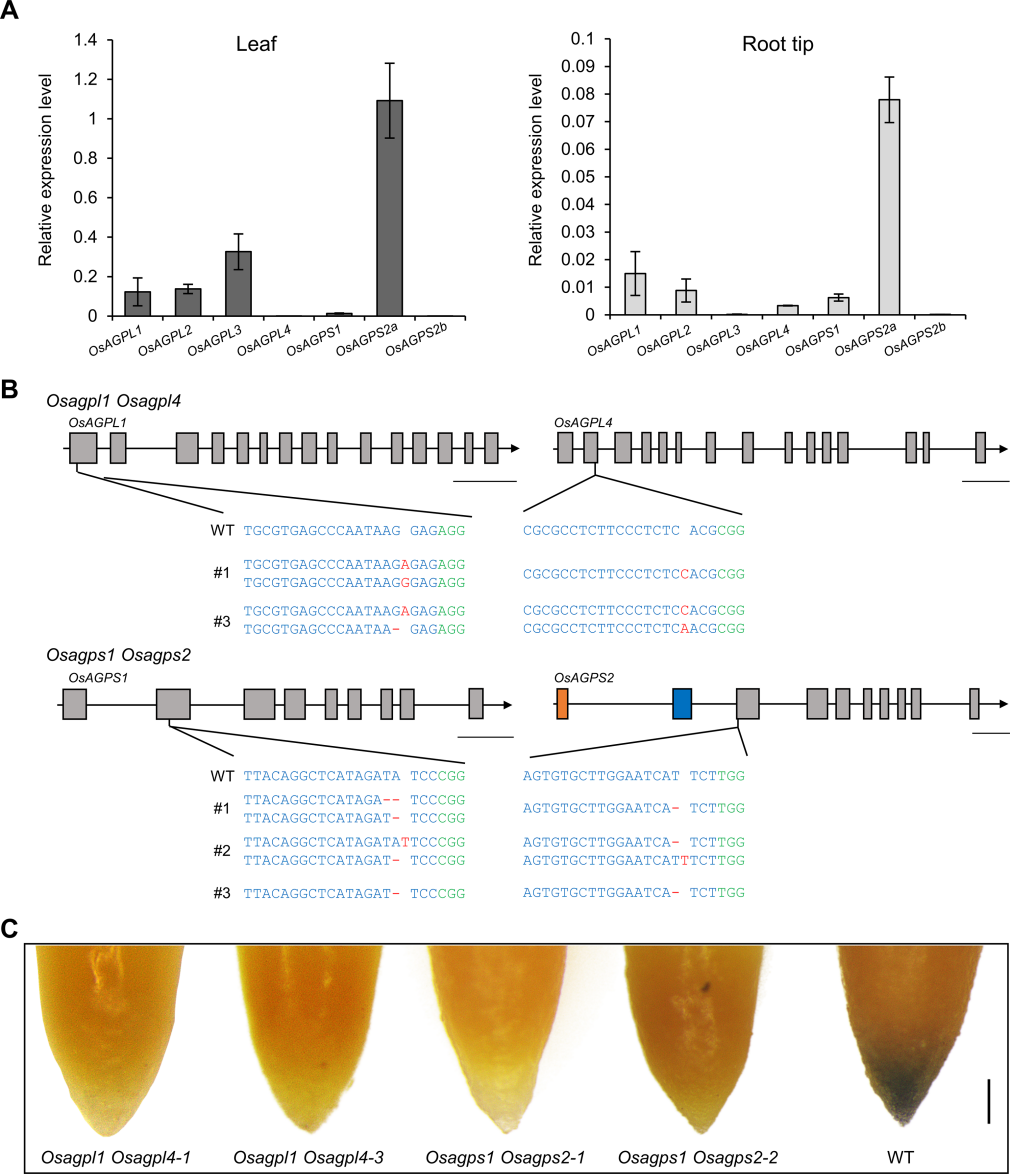
Expression analysis and genetic validation of plastidic AGPase isoform pairs required for root-tip starch biosynthesis. **(A)** RT-qPCR of AGPase gene expression in rice leaves and root tips. Transcript levels were normalized to *OsUBQ5*. Error bars represent SE. *n* = 4 biological replicates. **(B)** Schematic representation of CRISPR/Cas9 target sites and representative mutation profiles for *Osagpl1 Osagpl4* and *Osagps1 Osagps2* mutants. Target sequences (blue), protospacer-adjacent motifs (PAMs; green), and indel positions (red) are indicated. Exons are represented as boxes and introns as lines. For *OsAGPS2*, which produces two distinct transcripts, exon 1a and exon 1b are indicated by blue and orange boxes, respectively. Scale bars = 0.5 kb. **(C)** Representative images of Lugol’s iodine staining of root tips from wild-type (WT), *Osagpl1 Osagpl4*, and *Osagps1 Osagps2* double-knockout lines. Scale bar = 200 µm. Root-tip images were digitally extracted and arranged on a white background for clarity.

Because all single knockout mutants of AGPase genes retained normal starch accumulation in root tips, we hypothesized that multiple AGPase large and/or small subunits act redundantly in columella cells. To identify candidate genes, we analyzed *OsAGPL* and *OsAGPS* gene expression in root tips and leaves by RT-qPCR (**Figure 2A**). *OsAGPL1*, *OsAGPL2*, *OsAGPL4*, *OsAGPS1*, and *OsAGPS2a* transcripts were detected in root tips, whereas the expression of *OsAGPS2b*, which encodes a cytosolic protein, was undetectable. Importantly, *OsAGPL1*, *OsAGPL4*, *OsAGPS1*, and *OsAGPS2a* encode proteins predicted and experimentally shown to localize to plastids (Lee et al., 2007).

Based on these expression profiles and known subcellular localization, we reasoned that specific pairs of plastid-localized AGPase subunits, namely OsAGPL1 and OsAGPL4 for large subunits and OsAGPS1 and OsAGPS2a for small subunits, might be the principal contributors to starch biosynthesis in root-tip columella cells. To test this hypothesis, we generated double-knockout lines via CRISPR/Cas9-mediated gene editing targeting either *OsAGPL1* and *OsAGPL4* or *OsAGPS1* and *OsAGPS2* and selected transgenic rice plants carrying homozygous or biallelic mutations at the targeted loci that were predicted to generate frameshift-induced premature stop codons (**Figure 2B**; **Supplementary Figure 1**). For the predicted exonic off-target candidate sites of *OsAGPS1*, no mutations were detected at the analyzed off-target sites by Sanger sequencing (**Supplementary Table 3**; **Supplementary Figure 3**).

Lugol staining revealed a complete lack of starch accumulation in the root tips of two independent *Osagpl1 Osagpl4* lines (*Osagpl1 Osagpl4–1* and *Osagpl1 Osagpl4-2*) and two independent *Osagps1 Osagps2* lines (*Osagps1 Osagps2–1* and *Osagps1 Osagps2-2*) based on their clear or pale-yellow appearance. As a control, the root tips of WT plants exhibited strong staining (**Figure 2C**). These results indicate that plastidic AGPase function relies on two AGPase small subunits and two AGPase large subunits for starch biosynthesis in root-tip columella cells.

### 3.3 Isoform-specific analysis supports an exclusive requirement for plastidic AGPase subunits

**Figure 3:**
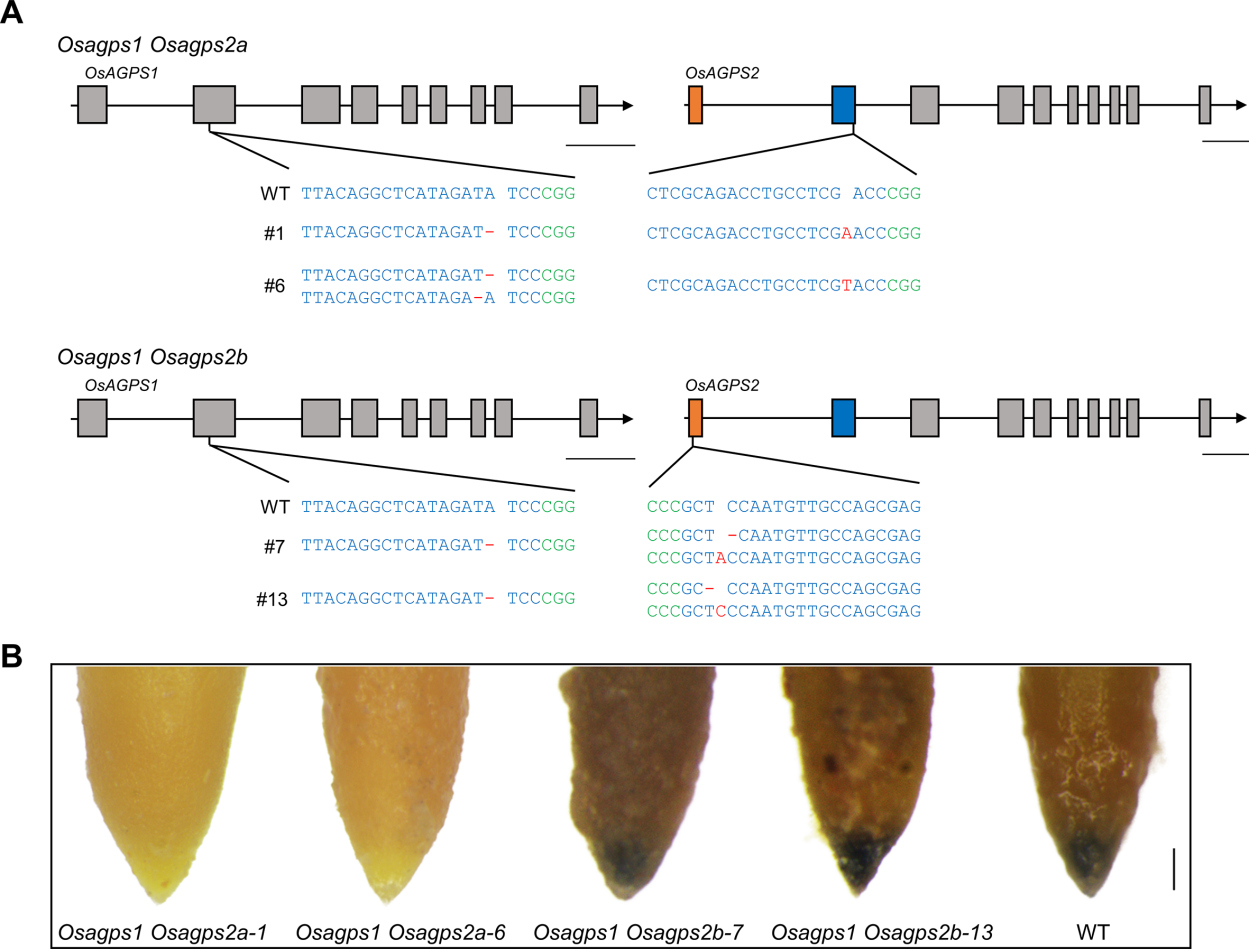
Isoform-specific requirement of *OsAGPS2a* for root-tip starch biosynthesis. **(A)** Schematic representation of CRISPR/Cas9 target sites and representative mutation profiles for *Osagps1 Osagps2a* and *Osagps1 Osagps2b* mutants. *OsAGPS1* was targeted at exon 2 in both mutant backgrounds, whereas isoform-specific sgRNAs were designed to target the unique first exon of either *OsAGPS2a* or *OsAGPS2b*. Target sequences (blue), protospacer-adjacent motifs (PAMs; green), and indel positions (red) are indicated. Exons are represented as boxes and introns as lines. For *OsAGPS2*, which produces two distinct transcripts, exon 1a and exon 1b are indicated by blue and orange boxes, respectively. Scale bars = 0.5 kb. **(B)** Representative images of Lugol’s iodine staining of root tips from wild-type (WT), *Osagps1 Osagps2a*, and *Osagps1 Osagps2b* independent double-knockout lines. Scale bar = 200 µm. Root-tip images were digitally extracted and arranged on a white background for clarity.

Because *OsAGPL2* was expressed in root tips at approximately half the level of *OsAGPL1* (**Figure 2A**), we wondered whether AGPase subunits predicted to localize to the cytosol might support starch biosynthesis in the absence of plastidic subunits. To test this possibility, we generated double mutants of *OsAGPS1* and *OsAGPS2a* (encoding a plastid-localized subunit) or *OsAGPS2b* (encoding a cytosolic subunit) via CRISPR/Cas9-mediated splicing variant–specific editing of the *OsAGPS2* locus from which *OsAGPS2a* and *OsAGPS2b* are transcribed (Lee et al., 2007) (**Figure 3A**).

For *OsAGPS1*, we used the same sgRNA targeting the exon 2 region as for the single mutants described above. For the *OsAGPS2* locus, we designed two distinct sgRNAs that specifically disrupt the first exon of either *OsAGPS2a* or *OsAGPS2b*, allowing for selective inactivation of one transcript isoform while leaving the other one intact. Sanger sequencing of the target sites confirmed successful isoform-specific mutagenesis in the selected plants, generating frameshift-induced premature stop codons (**Figure 3A**; **Supplementary Figure 1**). No mutations were detected by Sanger sequencing at the analyzed exonic off-target candidate sites of *OsAGPS1* (**Supplementary Table 3**; **Supplementary Figure 3**).

The two groups of mutants were clearly distinguished based on the intensity of Lugol staining (**Figure 3B**). Two independent mutant lines of *OsAGPS1* and *OsAGPS2a* (*Osagps1 Osagps2a-2* and *Osagps1 Osagps2a-6*) completely lacked starch accumulation, phenocopying the *Osagpl1 Osagpl4* and *Osagps1 Osagps2* double mutants. By contrast, the root tips of two independent *Osagps1 Osagps2b* lines (*Osagps1 Osagps2b-7* and *Osagps1 Osagps2b-13*) showed a normal staining signal and were morphologically indistinguishable from WT. These results demonstrate that starch biosynthesis in root tips requires the plastidic small subunit OsAGPS2a, whereas the cytosolic subunit OsAGPS2b is dispensable for this process.

### 3.4 OsGPT1 supplies glucose-6-phosphate for root-tip starch biosynthesis

Having identified the plastidic AGPase subunits required for starch biosynthesis, we sought to determine how carbon substrates are supplied to columella amyloplasts. In plastids, Glc-6-P is converted to Glc-1-P by phosphoglucomutase and subsequently used by AGPase to produce ADP-Glc (Lee et al., 2016). In rice, plastidic Glc-6-P import is mediated by members of the GPT family, including OsGPT1 and the three OsGPT2 paralogs: OsGPT2-1, OsGPT2-2, and OsGPT2-3. Because *OsGPT2–1* and *OsGPT2–2* share highly conserved coding sequences and exhibit similar expression profiles, we examined these two paralogs together in subsequent gene expression and functional assays (Toyota et al., 2006; Zhang et al., 2021).

**Figure 4:**
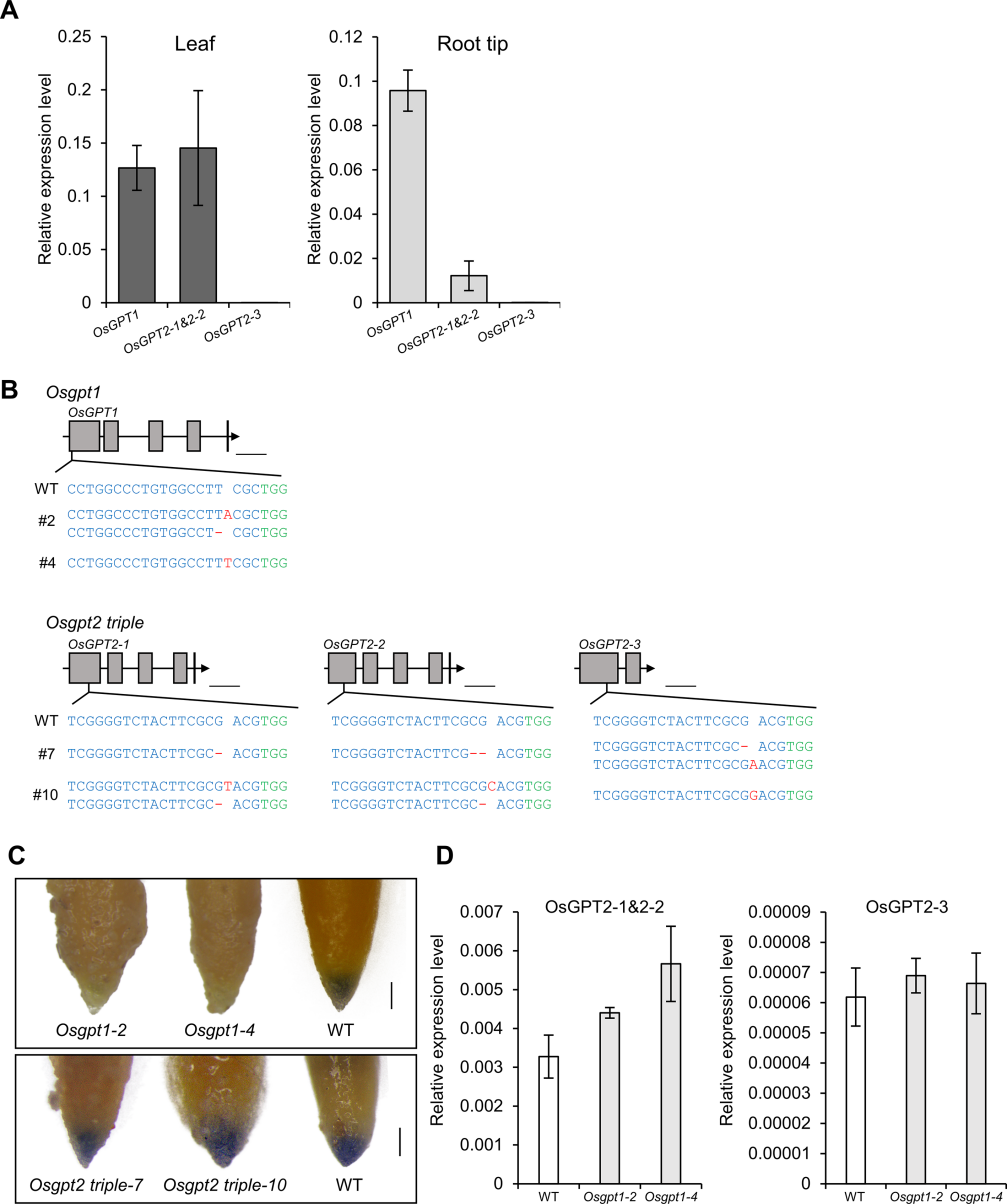
OsGPT1-dependent Glc-6-P supply for root-tip starch biosynthesis. **(A)** RT-qPCR of *OsGPT* gene expression in rice leaves and root tips. Transcript levels were normalized to *OsUBQ5*. Error bars represent SE. *n* = 4 biological replicates. **(B)** Schematic representation of CRISPR/Cas9 target sites and representative mutation profiles for *Osgpt1* and *Osgpt2* triple mutants. Target sequences (blue), protospacer-adjacent motifs (PAMs; green), and indel positions (red) are indicated. Exons are represented as boxes and introns as lines. For OsGPT2 paralogs, a single sgRNA targeting conserved sequences was used to simultaneously edit *OsGPT2-1*, *OsGPT2-2*, and *OsGPT2-3*. Scale bars = 0.5 kb. **(C)** Representative images of Lugol’s iodine staining of root tips from wild-type (WT), *Osgpt1* knockout, and *Osgpt2* triple-knockout lines. Scale bars = 200 µm. Root-tip images were digitally extracted and arranged on a white background for clarity. **(D)** RT-qPCR of the expression of *OsGPT2* paralogs in wild-type and *Osgpt1* root tips. Error bars represent SE. *n* = 4 biological replicates.

RT-qPCR revealed strong *OsGPT1* expression in both root tips and leaves, whereas the combined expression of *OsGPT2–1* and *OsGPT2–2* was low in root tips but high in leaves (**Figure 4A**), suggesting that OsGPT1 is the predominant active Glc-6-P transporter in columella cells. By contrast, *OsGPT2-3*, which is thought to be a truncated nonfunctional pseudogene, showed no detectable expression in either root tips or leaves under our conditions (Zhang et al., 2021; Qu et al., 2021).

To determine the functional contribution of *OsGPT1* and *OsGPT2* paralogs to root-tip starch accumulation, we analyzed CRISPR/Cas9-generated homozygous or biallelic mutant lines: *Osgpt1–2* and *Osgpt1–4* for *OsGPT1* and *Osgpt2 triple-7* and *Osgpt2 triple-10* for *OsGPT2-1*, *OsGPT2-2*, and *OsGPT2-3* (**Figure 4B**). Predicted amino acid sequence analysis indicated that these mutations resulted in truncated protein products (**Supplementary Figure 4**). Functional analysis using CRISPR/Cas9-generated mutants showed that the disruption of OsGPT1 function in the *Osgpt1–2* and *Osgpt1–4* single mutants resulted in much weaker Lugol staining in mutant root tips than in WT root tips (**Figure 4C**). By contrast, triple-knockout mutants of *OsGPT2*s, *Osgpt2 triple-7* and *Osgpt2 triple-10*, showed a normal staining pattern that was indistinguishable from that of WT. RT-qPCR of *OsGPT2-1*, *OsGPT2-2*, and *OsGPT2–3* expression in WT and *Osgpt1* mutants revealed no consistent or significant upregulation (**Figure 4D**), indicating that transcriptional compensation by *OsGPT2* paralogs is unlikely. These results indicate that OsGPT1 is required for efficient Glc-6-P import to support starch biosynthesis in columella amyloplasts, whereas OsGPT2 paralogs do not make a detectable contribution to this process.

### 3.5 Anatomical validation, starch and soluble sugar measurements, and an integrative model for starch biosynthesis in root-tip columella cells

**Figure 5:**
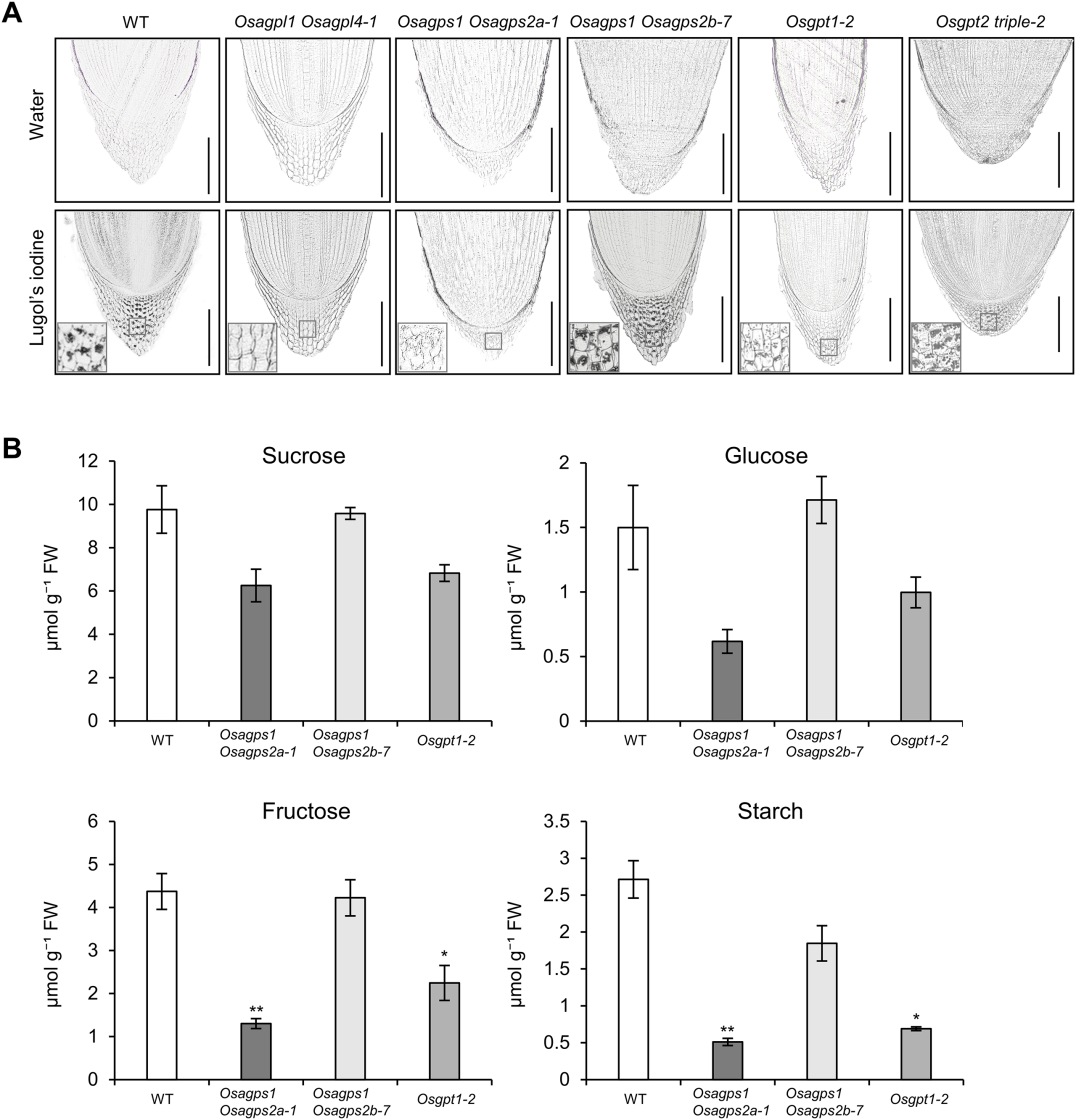
Histological validation and measurement of soluble sugar and starch contents in root tips. **(A)** Representative images of water-treated and Lugol’s iodine-stained resin-embedded longitudinal root-tip sections from wild-type (WT) and mutant seedlings (O*sagpl1 Osagpl4*, *Osagps1 Osagps2a*, *Osagps1 Osagps2b*, *Osgpt1*, and *Osgpt2* triple-knockout). Scale bars = 200 µm. Water-treated and Lugol’s iodine-stained images were obtained from the same root tip for each genotype. The root-tip areas corresponding to the sections analyzed were digitally extracted and arranged for comparison. For *Osagps1 Osagps2b*, the water-treated image was unavailable, and a representative image from a different root tip of the same genotype was used. Insets show magnified views of the boxed regions. **(B)** Starch and soluble sugar (sucrose, glucose, and fructose) contents in root-tip tissues of wild-type (WT), *Osagps1 Osagps2a*, *Osagps1 Osagps2b*, and *Osgpt1* seedlings collected at ZT5. Error bars represent SE. *n* = 3 biological replicates. * and ** indicate significant differences compared with WT, as determined by *t*-test (*P* < 0.05 and *P* < 0.01, respectively).

To further validate our observations in the mutants at the tissue level, we performed anatomical and metabolic analyses of starch biosynthesis in root tips. To anatomically validate the Lugol staining phenotypes observed in intact roots (**Figures 1**–**4**), we examined resin-embedded root-tip sections stained with iodine (**Figure 5A**). In WT root tip sections, columella amyloplasts were intensely stained, confirming abundant starch accumulation. Root tip sections from *Osagpl1 Osagpl4* and *Osagps1 Osagps2a* plants lacked detectable staining, whereas those from *Osagps1 Osagps2b* plants exhibited a staining comparable to that of the WT.

To assess the metabolic consequences of specific AGPase subunits, we measured the contents of starch and soluble sugars, including sucrose, glucose, and fructose, in root-tip tissues of WT and representative *Osagps1 Osagps2a* and *Osagps1 Osagps2b* mutant lines (**Figure 5B**). Starch contents were drastically lower in the root tips of *Osagps1 Osagps2a* seedlings than the WT, dropping from approximately 2.7 μmol g^−1^ FW in WT to 0.51 μmol g^−1^ FW in the mutant. Fructose levels were also significantly lower in mutant root tips. Similarly, sucrose and glucose levels appeared to be lower in the mutants, although these differences were not significant (**Figure 5B**). By contrast, metabolite levels in *Osagps1 Osagps2b* root tips were largely comparable to those of the WT, with no significant difference in starch content. Notably, the coordinated decline in starch and soluble sugar contents in *Osagps1 Osagps2a* root tips suggests a diminished sink strength in this tissue. Given that plastidic starch biosynthesis is a major determinant of carbon utilization and sink capacity, disrupting the plastidic AGPase small subunit likely attenuated the sucrose unloading gradient, thereby limiting the influx of photoassimilates into root-tip cells of the mutant. These results further support the observation that disrupting the functions of OsAGPS1 and OsAGPS2a, but not OsAGPS1 or OsAGPS2b, severely impaired starch biosynthesis in root-tip columella cells. This observation is consistent with the Lugol staining patterns described above and supports the functional specificity of the plastidic AGPase subunits OsAGPS1 and OsAGPS2a.

Notably, in root tip sections from *Osgpt1*, we observed weak and patchy staining, which is consistent with much lower, although not completely abolished, starch accumulation (**Figure 5A**). By contrast, root tip sections from the *Osgpt2*-*triple* mutant were indistinguishable from those of the WT. These histological observations corroborate the results of genetic analysis and whole-root staining.

To further assess the biochemical basis of the *Osgpt1* mutant phenotype, we analyzed starch and soluble sugar contents in the root tips of WT and *Osgpt1–2* plants (as a representative *Osgpt1* mutant; **Figure 5B**). *Osgpt1–2* root tips accumulated much less starch than the WT, reaching approximately 0.69 μmol g^−1^ FW. However, the starch level of *Osgpt1–2* root tips was slightly higher than that of *Osagps1 Osagps2a* root tips (**Figure 5B**). The presence of residual starch accumulation, together with a higher starch level compared to *Osagps1 Osagps2a*, suggests that limited carbon flux into plastids may still occur through minor or alternative transport routes, even in the absence of OsGPT1. In the soluble sugar fraction, fructose content was significantly lower in *Osgpt1–2* than in WT, whereas sucrose and glucose levels were not significantly different from the WT. These results indicate that the loss of OsGPT1 function strongly impairs starch accumulation in root tips and is accompanied by a shift in soluble sugar composition.

**Figure 6:**
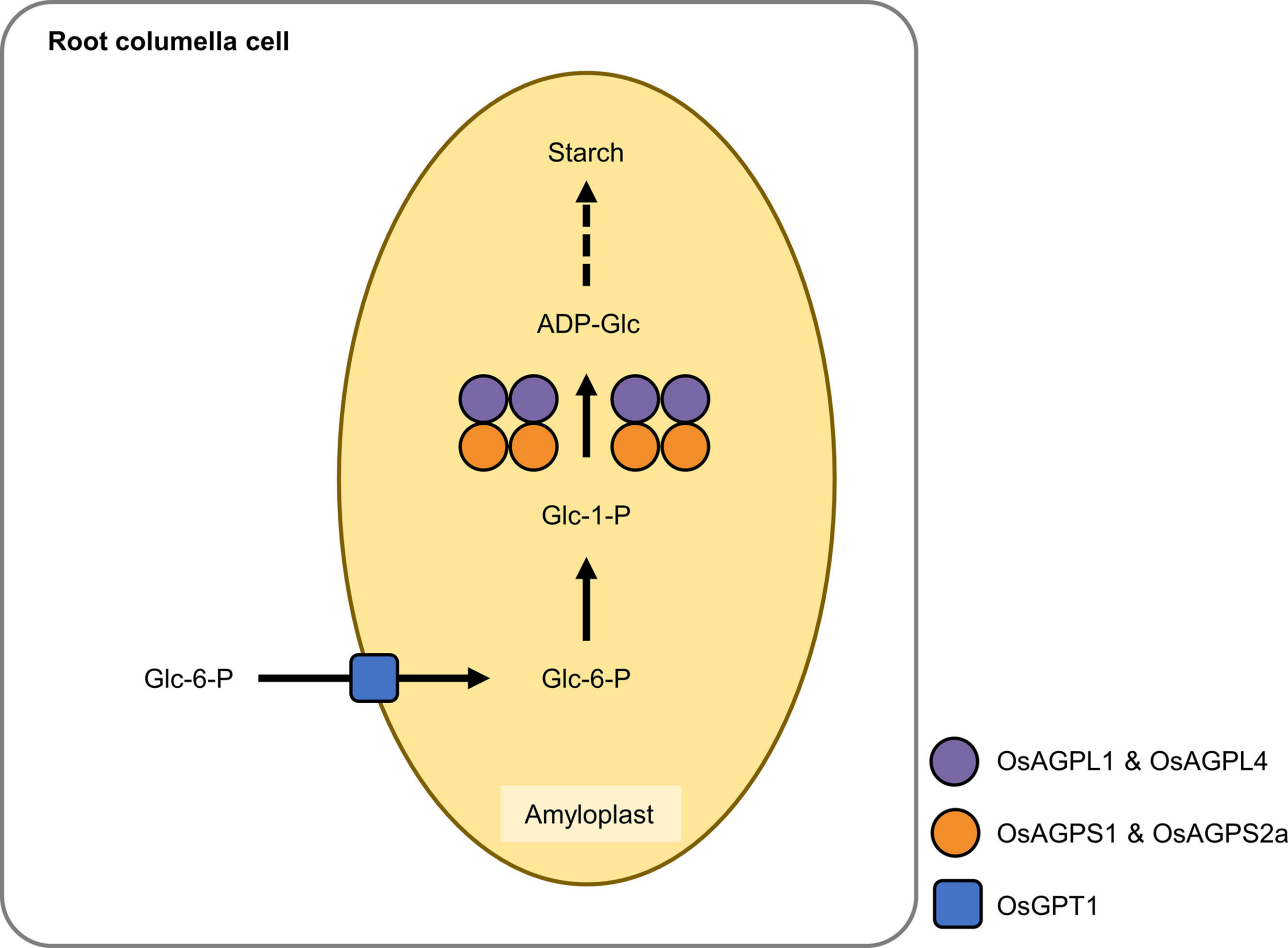
A model of starch biosynthesis in root-tip columella cells. Schematic model of starch biosynthesis in columella cells in rice root tips. Glucose-6-phosphate (Glc-6-P) is predominantly imported into columella amyloplasts via the plastidial Glc-6-P/phosphate translocator OsGPT1. Inside the amyloplast, Glc-6-P is converted to glucose-1-phosphate (Glc-1-P) by plastidic phosphoglucomutase (PGM), and ADP-glucose (ADP-Glc) is synthesized by a plastidic AGPase complex composed of the large-subunit isoforms OsAGPL1 and OsAGPL4 and the small-subunit isoforms OsAGPS1 and OsAGPS2a. The resulting ADP-Glc is then utilized for starch biosynthesis, establishing a plastid-localized starch biosynthetic pathway distinct from the cytosolic route operating in cereal endosperm. Different colors indicate distinct AGPase subunit classes and associated components included in the model.

Based on these results, we propose an integrative model for starch biosynthesis in rice root-tip columella cells (**Figure 6**). According to this model, Glc-6-P is imported into columella amyloplasts predominantly via OsGPT1, converted to Glc-1-P by phosphoglucomutase, and subsequently utilized by a plastidic AGPase system that requires plastidic AGPase subunits, including OsAGPL1, OsAGPL4, OsAGPS1, and OsAGPS2a, to generate ADP-Glc for starch biosynthesis. Although plastidic AGPase is expected to function as an L_2_S_2_ heterotetramer, the precise subunit combinations operating in root-tip columella cells remain unresolved. This plastid-localized pathway explains the phenotypes of all mutant classes and defines a distinct starch biosynthetic system operating in root-tip columella cells.

## 4 Discussion

### 4.1 A plastidic starch biosynthetic pathway operates specifically in rice root-tip columella cells

In this study, we systematically defined the starch biosynthetic pathway operating in rice root-tip columella cells and demonstrated that it depends exclusively on plastid-localized components. Through a combination of CRISPR/Cas9-mediated mutagenesis, Lugol’s iodine staining–based screening, gene expression analysis, anatomical validation, and analysis of soluble sugar and starch contents (**Figures 1**-**5**), we showed that starch accumulation in columella amyloplasts requires the plastid-localized AGPase subunits OsAGPL1, OsAGPL4, OsAGPS1, and OsAGPS2a, together with the Glc-6-P transporter OsGPT1. Cytosolic AGPase subunits and OsGPT2 paralogs are dispensable for this pathway.

These findings establish that rice root-tip columella cells utilize a fully plastidic starch biosynthetic route, in which Glc-6-P is imported into amyloplasts and locally converted to ADP-Glc (**Figure 6**). This pathway is mechanistically distinct from the cytosolic AGPase-dependent route operating in developing endosperm (Lee et al., 2007; Li et al., 2017; Huang et al., 2021), reinforcing the notion that starch biosynthesis in rice is organized into organ-specific metabolic modules.

### 4.2 Organ-specific deployment of AGPase subunits underlies metabolic specialization in rice

Previous studies in rice have revealed strong tissue specificity in AGPase function. In the endosperm, cytosolic AGPase plays a dominant role in generating ADP-Glc for massive starch deposition during grain filling (Lee et al., 2007; Tang et al., 2016; Huang et al., 2021), whereas plastid-localized AGPase contributes little to this process. By contrast, starch accumulation in vegetative sink tissues (such as stems) predominantly depends on plastidic AGPase, particularly the large subunit OsAGPL1 (Hirose et al., 2006; Okamura et al., 2013). Similarly, starch biosynthesis in pollen relies on plastid-localized hexose phosphate metabolism rather than cytosolic ADP-Glc production (Lee et al., 2016).

Our results extend this framework to the root tip. Expression analysis identified *OsAGPL1*, *OsAGPL4*, *OsAGPS1*, and *OsAGPS2a* as the predominant AGPase subunit genes expressed in root tips (**Figure 2A**). Genetic analysis demonstrated that simultaneously disrupting both large or both small plastidic AGPase subunits abolished columella starch accumulation (**Figures 2C**, **3B**). Notably, despite detectable expression of *OsAGPL2* (encoding a cytosolic subunit), loss of function of the plastid-localized small subunits OsAGPS1 and OsAGPS2a completely eliminated starch production (**Figure 3B**). This finding indicates that cytosolic AGPase function cannot compensate for the loss of plastidic AGPase in columella cells and that ADP-Glc produced in the cytosol is not readily accessible to the plastid-localized starch biosynthetic machinery.

Together, these findings support a model in which rice deploys distinct AGPases in different tissues to establish metabolically insulated starch pools, allowing flexible, independent regulation of carbon partitioning across tissues (Hirose et al., 2006; Okamura et al., 2013; Tang et al., 2016; Lee et al., 2016).

### 4.3 OsGPT1 defines the carbon entry point for columella amyloplast metabolism

Our identification of OsGPT1 as the primary Glc-6-P transporter supplying substrate for starch biosynthesis in columella cells provides a critical mechanistic link between cytosolic carbon metabolism and plastidic starch biosynthesis in the root tip. *OsGPT1* was strongly expressed in root tips, whereas *OsGPT2* paralogs showed minimal expression (**Figure 4A**). Moreover, functional analysis revealed that loss of OsGPT2 function had no detectable effect on starch accumulation in root tips (**Figure 4C**).

By contrast, *Osgpt1* mutants showed much weaker Lugol staining in root tips (**Figure 4C**). Analysis of root tip sections confirmed that starch accumulation was much lower in the mutants than the WT but not completely abolished in columella amyloplasts (**Figure 5A**). Notably, this residual starch accumulation was only weakly detected by conventional Lugol staining and whole root-tip enzymatic assays, as *Osgpt1* root tips showed only subtle differences compared with *Osagpl1 Osagpl4* and *Osagps1 Osagps2a* root tips, likely reflecting methodological sensitivity and anatomical constraints. Indeed, previous studies have suggested that conventional Lugol staining may underestimate very low levels of starch; in fact, higher-sensitivity optical imaging approaches have shown that samples that appear nearly starchless by routine Lugol staining can still retain small starch granules (Smith and Zeeman, 2006; Ovecka et al., 2012; Gala De Pablo et al., 2023). Moreover, because our biochemical measurements were performed using whole root tips rather than isolated columella cells, the contribution of starch-rich columella amyloplasts was likely diluted by surrounding non-starchy cell types, thereby diminishing the apparent magnitude of differences among genotypes (Moussaieff et al., 2013; Zheng et al., 2024). This effect may also explain why absolute starch values remained relatively low even in the root tips of WT plants. The residual starch staining observed in the *Osgpt1* mutant suggests that limited carbon input from alternative routes, including through OsGPT2 paralogs, minor hexose phosphate transport via other plastidic transporters, or metabolic recycling within plastids, may sustain some starch biosynthesis in the absence of OsGPT1. However, the lack of upregulation of *OsGPT2* paralogs (**Figure 4D**) indicates that OsGPT1 likely represents the primary and physiologically relevant Glc-6-P import route in columella cells.

This situation closely parallels that reported for Arabidopsis, in which GPT1 is essential for columella starch biosynthesis (Niewiadomski et al., 2005; Tsai et al., 2009), suggesting that plastidic Glc-6-P import is a conserved feature of root-cap starch metabolism across dicots and monocots, despite broader divergence in starch biosynthetic strategies among plant organs.

### 4.4 Columella starch serves as a regulated root sink

Although columella starch has classically been studied in the context of gravity perception (Perrin et al., 2005; Kawamoto and Morita, 2022), our results highlight the regulation of its metabolism. The strict requirement for plastidic AGPase and OsGPT1 indicates that starch accumulation in columella cells is not a passive consequence of carbon availability but rather reflects an actively regulated sink activity within the root apex (**Figures 3**-**5**). In this context, the coordinated drop in starch and soluble sugar levels observed in mutants lacking plastidic AGPase or OsGPT1 further supports the view that columella starch biosynthesis contributes to local sink strength. Disrupting plastidic carbon utilization likely attenuates the sucrose unloading gradient, thereby limiting the influx of photoassimilates into root-tip cells. These findings suggest that starch biosynthesis in columella amyloplasts does not merely contribute to starch storage but functions as a key metabolic driver of sink activity at the root apex.

The root tip represents a highly dynamic metabolic zone, characterized by rapid cell turnover and continuous interactions with the soil environment. Establishing a localized starch pool in columella amyloplasts may provide buffering capacity against transient fluctuations in carbon supply or may help support sustained metabolic activity under suboptimal conditions (Thalmann and Santelia, 2017). The modular organization of this pathway suggests that columella starch metabolism can be independently regulated without perturbing starch biosynthesis in other organs.

Notably, despite the severe reduction of columella starch accumulation, the AGPase double mutants, *Osagpl1 Osagpl4* and *Osagps1 Osagps2*, which lacked starch accumulation in root tips, did not exhibit obvious defects in overall root-system morphology under our experimental conditions (**Supplementary Figure 5**). This suggests that plastidic starch biosynthesis in columella cells is not strictly required for bulk root growth during normal seedling establishment but instead may play more specialized roles in local metabolic buffering, gravitropic function, or adaptation to fluctuating environmental conditions. The absence of strong morphological phenotypes also indicates that compensatory carbon metabolic pathways may partially sustain root growth outside the columella starch pathway.

### 4.5 Implications for localized root carbon metabolism

From an applied perspective, the genetic definition of a plastidic starch biosynthetic pathway specific to root-tip columella cells provides a useful framework for exploring how localized carbon metabolism contributes to root function. Unlike starch biosynthesis in the endosperm, which is tightly linked to yield and often associated with pleiotropic effects, the columella pathway defined here appears to represent a more spatially restricted and metabolically specialized system.

Targeted modulation of OsGPT1 activity or plastidic AGPase function could, in principle, alter carbon allocation to the root tip during early seedling establishment or under environmental conditions that place high metabolic demands on the root apex. Although the physiological consequences of such manipulation remain to be explored, our findings establish a mechanistic foundation for future studies aimed at optimizing carbon metabolism in roots to improve plant performance and resilience (Ghorbanzadeh et al., 2023).

### 4.6 Integrated organ-level model of starch biosynthesis in rice

Collectively, our results, together with previous findings, support an integrated model in which starch biosynthesis in rice is organized through the organ-specific deployment of carbon entry routes and combinations of small and large AGPase subunits. In photosynthetic leaves, carbon fixed via the Calvin–Benson–Bassham cycle is directly converted to starch through the activity of AGPase comprising OsAGPS2a and OsAGPL3 within chloroplasts (Lee et al., 2007). By contrast, the developing endosperm predominantly utilizes cytosolic AGPase consisting of OsAGPS2b and OsAGPL2 to generate ADP-Glc, which is subsequently transported into amyloplasts to sustain high-capacity starch accumulation under hypoxic conditions (Lee et al., 2007; Ohdan et al., 2005; Tuncel et al., 2014; Lee and Jeon, 2020). In vegetative sink tissues such as stems, starch biosynthesis primarily depends on plastidic AGPase activity, with OsAGPL1 functioning as a major large subunit, whereas the corresponding small subunit(s) remain unresolved (Cook et al., 2012; Okamura et al., 2013). In pollen grains, starch biosynthesis relies on plastidic hexose phosphate metabolism in which Glc-6-P is imported into amyloplasts and converted to ADP-Glc, resembling leaf-type plastidic pathways and relying on the large subunit AGPL4; the corresponding AGPS is currently unknown (Lee et al., 2016).

Our findings extend this framework to root-tip columella cells, demonstrating that starch biosynthesis in this tissue also follows a plastidic pathway dependent on Glc-6-P import via OsGPT1 and the plastidic AGPase subunits AGPL1, AGPL4, AGPS1, and AGPS2a (**Figure 6**). Notably, although both pollen grains and root tissues employ plastidic pathways, the specific AGPase isoform and regulatory contexts differ, reflecting tissue-specific metabolic demands.

Together, these observations support a unified model in which starch biosynthesis in rice is organized through modular, organ-specific metabolic configurations, allowing for independent regulation of carbon partitioning among source tissues, storage organs, and specialized sink tissues such as pollen grains and root tips. This integrated view highlights the flexibility of starch metabolism and provides a conceptual framework for understanding how plants optimize carbon allocation across distinct developmental and physiological contexts.

## Data Availability

The original contributions presented in the study are included in the article/**Supplementary Material**. Further inquiries can be directed to the corresponding author.

## Supplementary material

Supplementary Table 1 Summary of sgRNA target sequences and genome-wide match counts.

Supplementary Table 2 List of PCR primers used in this study.

Supplementary Table 3 Predicted off-target candidates identified by Cas-OFFinder.

Supplementary Figure 1 Predicted OsAGPase protein sequences encoded by CRISPR/Cas9-induced mutant alleles. Predicted amino acid sequences of AGPase proteins in single mutants **(A)** and multiple mutants **(B)**. Yellow boxes indicate wild-type-derived amino acid sequences, whereas cyan boxes indicate mutant-specific amino acid sequences generated after frameshift mutations. Dots indicate omitted amino acid residues, and asterisks indicate stop codons. The numbers correspond to the amino acid positions immediately before and after the mutation-derived sequence and the position of the last amino acid residue in the truncated protein.

Supplementary Figure 2 Length of the Lugol-positive region in root-tip columella cells measured from Lugol-stained root tips of WT and single AGPase knockout mutants. Data represent mean ± SD. WT, *n* = 9; single mutants, *n* = 3 each. Statistical analysis was performed using one-way ANOVA followed by Tukey’s HSD test. Different letters indicate statistically significant differences (*P* < 0.05). No statistically significant differences were detected among the analyzed genotypes. Although *Osagpl3* showed a relatively lower mean value, Lugol-positive starch accumulation was retained in all single-mutant lines.

Supplementary Figure 3 Representative Sanger sequencing chromatograms of predicted exonic off-target candidate sites for *OsAGPS1* CRISPR/Cas9 target sequences in the analyzed mutant lines. No detectable mutations were identified at the predicted off-target loci. Predicted off-target candidates were identified using Cas-OFFinder.

Supplementary Figure 4 Predicted OsGPT protein sequences encoded by CRISPR/Cas9-induced mutant alleles. Predicted amino acid sequences of OsGPT1 and OsGPT2 proteins in *Osgpt1* and *Osgpt2 triple* mutants. Yellow boxes indicate wild-type-derived amino acid sequences, whereas colored boxes indicate mutant-specific amino acid sequences generated after frameshift mutations. Dots indicate omitted amino acid residues, and asterisks indicate stop codons. The numbers correspond to the amino acid positions immediately before and after the mutation-derived sequence and the position of the last amino acid residue in the truncated protein.

Supplementary Figure 5 Representative root-system images of WT, *Osagpl1 Osagpl4*, and *Osagps1 Osagps2* plants. No obvious gross root morphological differences were observed between WT and the AGPase double-mutant lines under the examined conditions. Scale bar = 5 cm.
