# Supplementary material for: A plastidic starch biosynthetic pathway defines columella-specific carbon metabolism in rice root tips
Source: Front Plant Sci. 2026 Jun 1;17:1852363. doi: 10.3389/fpls.2026.1852363 (PMC13267343; doi:10.3389/fpls.2026.1852363)
Supplement: Supplementary Table 1 — Summary of sgRNA target sequences and genome-wide match counts. [file DataSheet1.pdf]

Supplementary Material

Supplementary Table S1. Summary of sgRNA target sequences and genome-wide match counts

Gene	20-nt spacer + PAM	20mer + PAM	12mer + PAM	8mer + PAM
OsAGPL1	TGCGTGAGCCCAATAAGGAGAGG	1	1	808
OsAGPL2	CTTGTGCACAACCAATGAGAAGG	1	1	885
OsAGPL3	TCAACTCCGCGTCGCTCAACCGG	1	1	986
OsAGPL4	CGCGCCTCTTCCCTCTCACGCGG	1	1	249
OsAGPS1	TTACAGGCTCATAGATATCCCGG	1	1	243
OsAGPS2	AGTGTGCTTGGAATCATTCTTGG	1	1	467
OsAGPS2a	CTCGCAGACCTGCCTCGACCCGG	1	1	472
OsAGPS2b	CCCGCTCCAATGTTGCCAGCGAG	1	1	745
OsGPT1	CCTGGCCCTGTGGCCTTCGCTGG	1	1	519
OsGPT2-1/2-2/2-3	TCGGGGTCTACTTCGCGACGTGG	3	3	652

Genome-wide match counts were obtained using the rice reference genome. The 12-nt and 8-nt sequences correspond to PAM-proximal seed-like regions. The conserved *OsGPT2* target sequence was intentionally designed to match *OsGPT2-1*, *OsGPT2-2*, and *OsGPT2-3*.

Supplementary Table S2. List of PCR primers used in this study

Primer name	Sequence (5' to 3')
OsAGPL1-qRT-F	CCTTCTTCACTTCACCTCGATATT
OsAGPL1-qRT-R	TCGATGATGCAGTTGTTTATCTTT
OsAGPL2-qRT-F	CGAGAACACAAAGATAAGGAACTG
OsAGPL2-qRT-R	CATTCTTCAGGATCACCACAAT
OsAGPL3-qRT-F	GGGGAGAATACAAAGATTCAGAAC
OsAGPL3-qRT-R	ATCGAGTTCTTTAATACGATGGTG
OsAGPL4-qRT-F	GATATGGGAACTTTTGGTTTG
OsAGPL4-qRT-R	AGATAGGCCTGCACATTGTA
OsAGPS1-qRT-F	TTATTAGACATTGCACAATCAACC
OsAGPS1-qRT-R	GTCAATTATTGCTTTTCTGATGTG
OsAGPS2a-qRT-F	TCCTCCTCCTCCTCCTCTTC
OsAGPS2a-qRT-R	CAGTTGCTGACAGGGATATCAA
OsAGPS2b-qRT-F	ACAAAAATCTTGACCGCAGTGT
OsAGPS2b-qRT-R	CTCCAAGAATGATTCCAAGCAC
OsGPT1-qRT-F	GCAAGCACTGAGGCCAATTTTG
OsGPT1-qRT-R	AAGGAACAAGAAACGAGCAACATAGAC
OsGPT2-1&2-2-qRT-F	CGGAACATATTCTCCAAGAAG
OsGPT2-1&2-2-qRT-R	ACACTTGGTTGTACAAGTGGTAG
OsGPT2-3-qRT-F	TTCTGGAAGGCCCTGTCA
OsGPT2-3-qRT-R	GAGGAAGAACCTGGAGACGA
OsUBQ5-qRT-F	GCACAAGCACAAGAAGGTGA
OsUBQ5-qRT-R	GCCTGCTGGTTGTAGACGTA

Supplementary Table S3. Predicted off-target candidates identified by Cas-OFFinder

Target gene	Predicted off-target sequence	Annotation	Validation
OsAGPL1	TGtGTcAGCCCAAaAAGGAGGGG	*Os09g0353200* intron	Non-coding candidate
OsAGPS2	AGTGTGgTTGGcATCtTTCTAGG	*Os08g0509500* intron	Non-coding candidate
OsAGPS2	AGTGTtCTTGGgATCATcCTTGG	*OsAGPS1* exon	No mutation detected
OsAGPS2a	CTtGCAGACCTGCCTCtgCCTGG	Intergenic region	Non-coding candidate
OsAGPS2a	CTCGCAGACgTGCCTCGAtCCGG	*OsAGPS1* exon	No mutation detected
OsAGPS2b	CTCGCTGGaAACAgaGGAGCTGG	Intergenic region	Non-coding candidate
OsAGPS2b	CTaGtTGGCAAaATTGGAGCTGG	Intergenic region	Non-coding candidate
OsGPT1	CCTGGCCtTGgGGCCTTgGCCGG	Intergenic region	Non-coding candidate

Predicted off-target candidates were identified using Cas-OFFinder against the rice reference genome. Nucleotides that are mismatched relative to the sgRNA target sequence are indicated by lowercase letters.

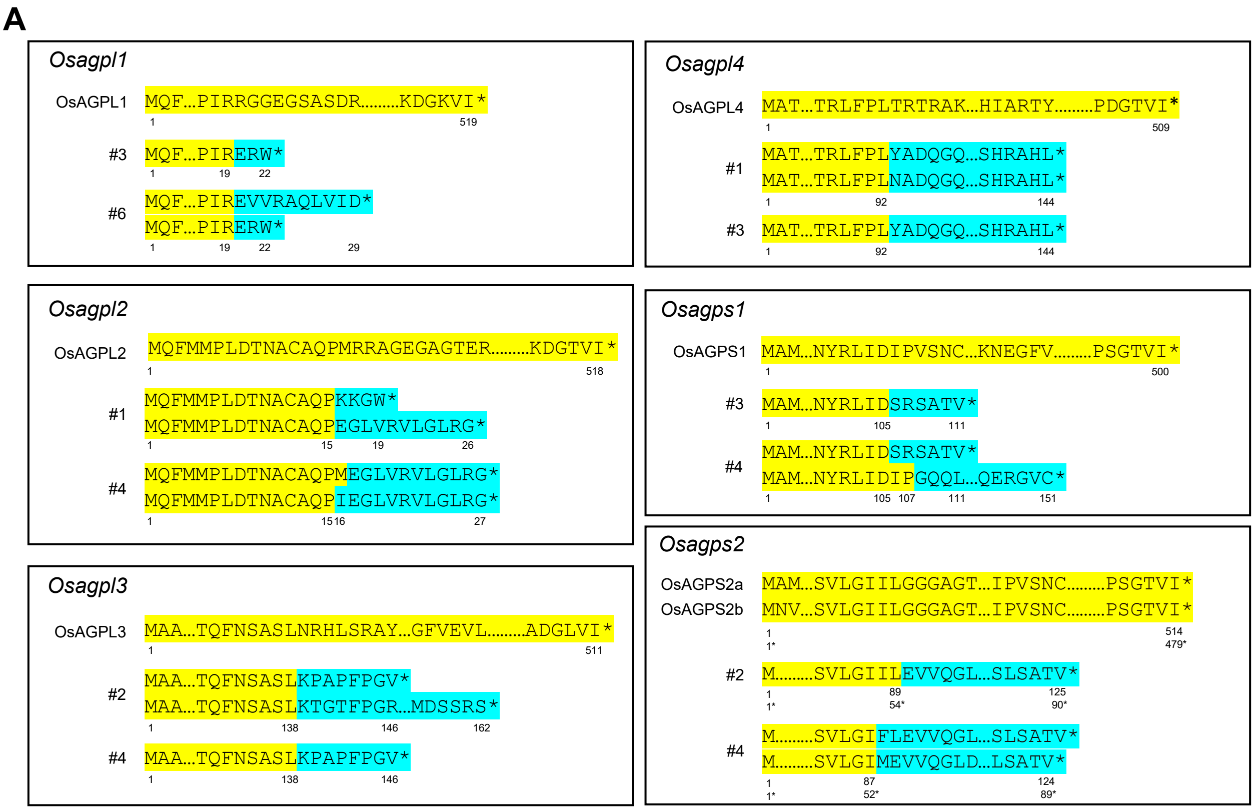
A
Osagpl1
OsAGPL1 MQF…PIRRGGEGSASDR………KDGKVI*
1 519
#3 MQF…PIRERW*
1 19 22
#6 MQF…PIREVVRAQLVID*
MQF…PIRERW*
1 19 22 29
Osagpl2
OsAGPL2 MQFMMPLDTNACAQPMRRAGEGAGTER………KDGTVI*
1 518
#1 MQFMMPLDTNACAQPKKGW*
MQFMMPLDTNACAQPEGLVRVLGLRG*
1 15 19 26
#4 MQFMMPLDTNACAQPMEGLVRVLGLRG*
MQFMMPLDTNACAQPIEGLVRVLGLRG*
1 15 16 27
Osagpl3
OsAGPL3 MAA…TQFNSASLNRHLSRAY…GFVEVL………ADGLVI*
1 511
#2 MAA…TQFNSASLKPAPFPGV*
MAA…TQFNSASLKTGTFPGR…MDSSRS*
1 138 146 162
#4 MAA…TQFNSASLKPAPFPGV*
1 138 146
Osagpl4
OsAGPL4 MAT…TRLFPLTRTRAK…HIARTY………PDGTVI*
1 509
#1 MAT…TRLFPLYADQGQ…SHRAHL*
MAT…TRLFPLNADQGQ…SHRAHL*
1 92 144
#3 MAT…TRLFPLYADQGQ…SHRAHL*
1 92 144
Osagps1
OsAGPS1 MAM…NYRLIDIPVSNC…KNEGFV………PSGTVI*
1 500
#3 MAM…NYRLIDSRSATV*
1 105 111
#4 MAM…NYRLIDSRSATV*
MAM…NYRLIDIPGQQL…QERGVC*
1 105 107 111 151
Osagps2
OsAGPS2a MAM…SVLGIILGGGAGT…IPVSNC………PSGTVI*
OsAGPS2b MNV…SVLGIILGGGAGT…IPVSNC………PSGTVI*
1 514
1* 479*
#2 M………SVLGIILEVVQGL…SLSATV*
1 89 125
1* 54* 90*
#4 M………SVLGIFLEVVQGL…SLSATV*
M………SVLGIMEVVQGLD…LSATV*
1 87 124
1* 52* 89*

B

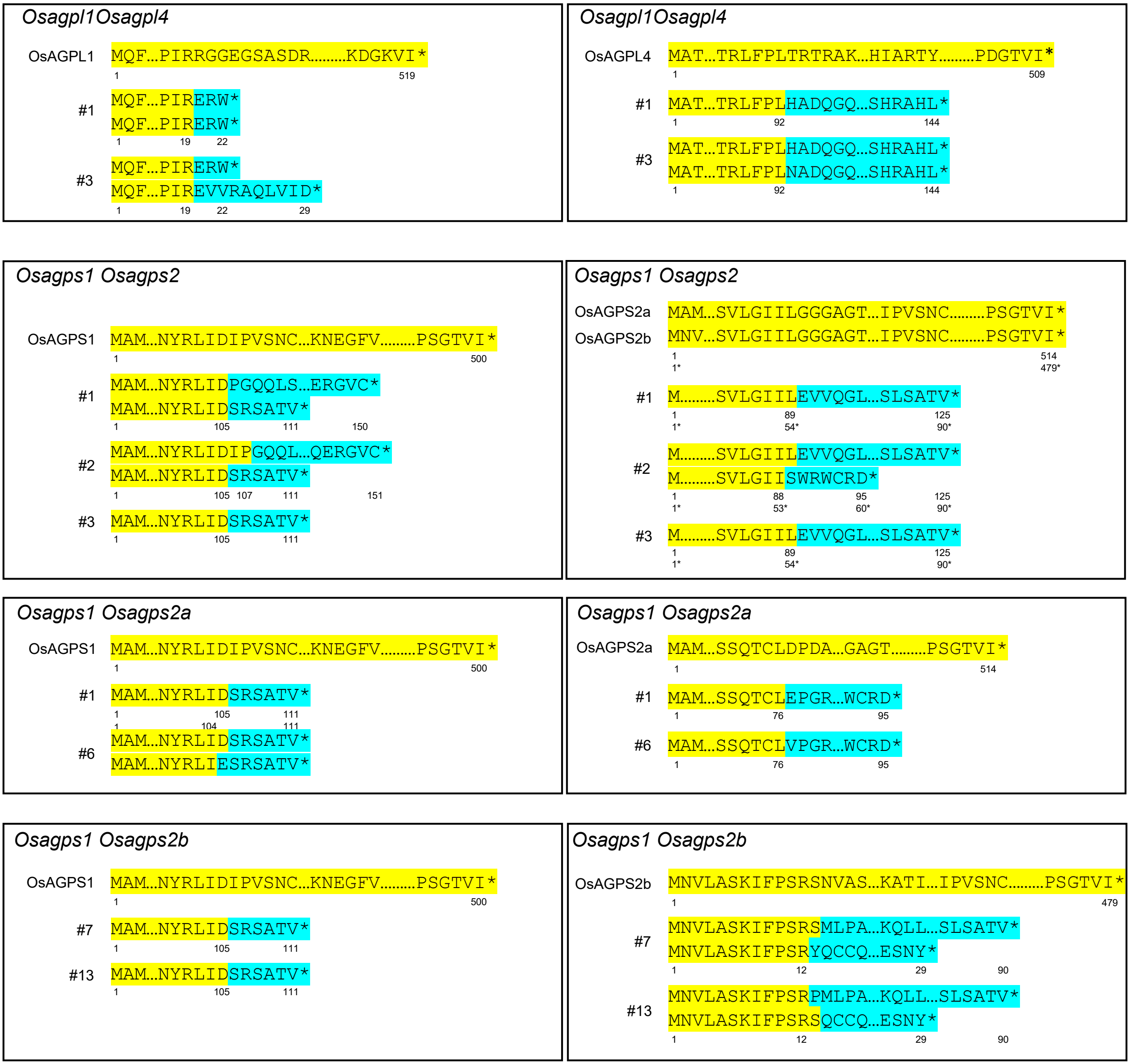

Supplementary Figure S1. Predicted OsAGPase protein sequences encoded by CRISPR/Cas9-induced mutant alleles. Predicted amino acid sequences of AGPase proteins in single mutants (A) and multiple mutants (B). Yellow boxes indicate wild-type-derived amino acid sequences, whereas cyan boxes indicate mutant-specific amino acid sequences generated after frameshift mutations. Dots indicate omitted amino acid residues, and asterisks indicate stop codons. The numbers correspond to the amino acid positions immediately before and after the mutation-derived sequence and the position of the last amino acid residue in the truncated protein.

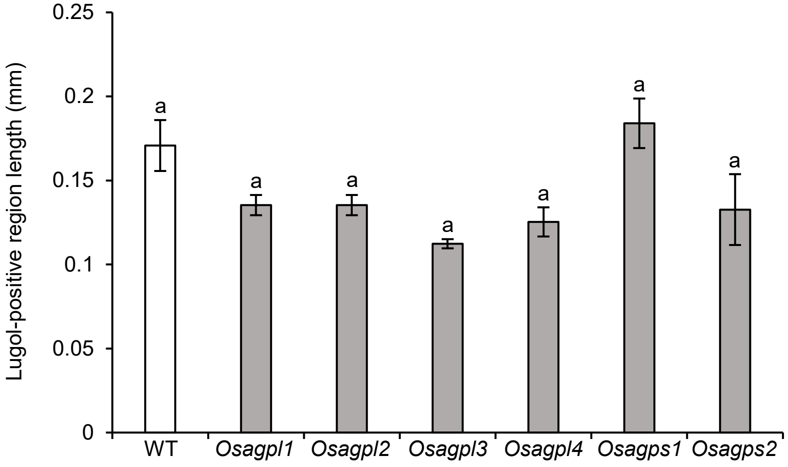

Supplementary Figure S2. Length of the Lugol-positive region in root-tip columella cells measured from Lugol-stained root tips of WT and single AGPase knockout mutants. Data represent mean ± SD. WT, $n = 9$; single mutants, $n = 3$ each. Statistical analysis was performed using one-way ANOVA followed by Tukey's HSD test. Different letters indicate statistically significant differences ($P < 0.05$). No statistically significant differences were detected among the analyzed genotypes. Although *Osagpl3* showed a relatively lower mean value, Lugol-positive starch accumulation was retained in all single mutant lines.

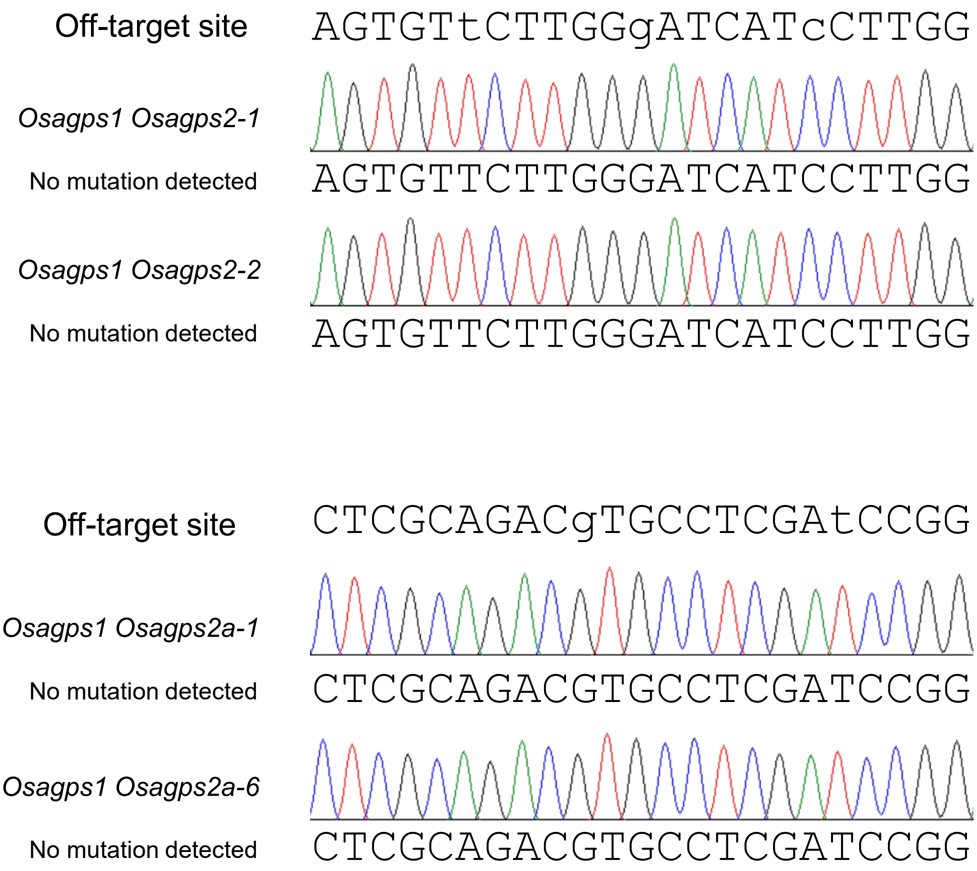

Supplementary Figure S3. Representative Sanger sequencing chromatograms of predicted exonic off-target candidate sites for *OsAGPS1* CRISPR/Cas9 target sequences in the analyzed mutant lines. No detectable mutations were identified at the predicted off-target loci. Predicted off-target candidates were identified using Cas-OFFinder.

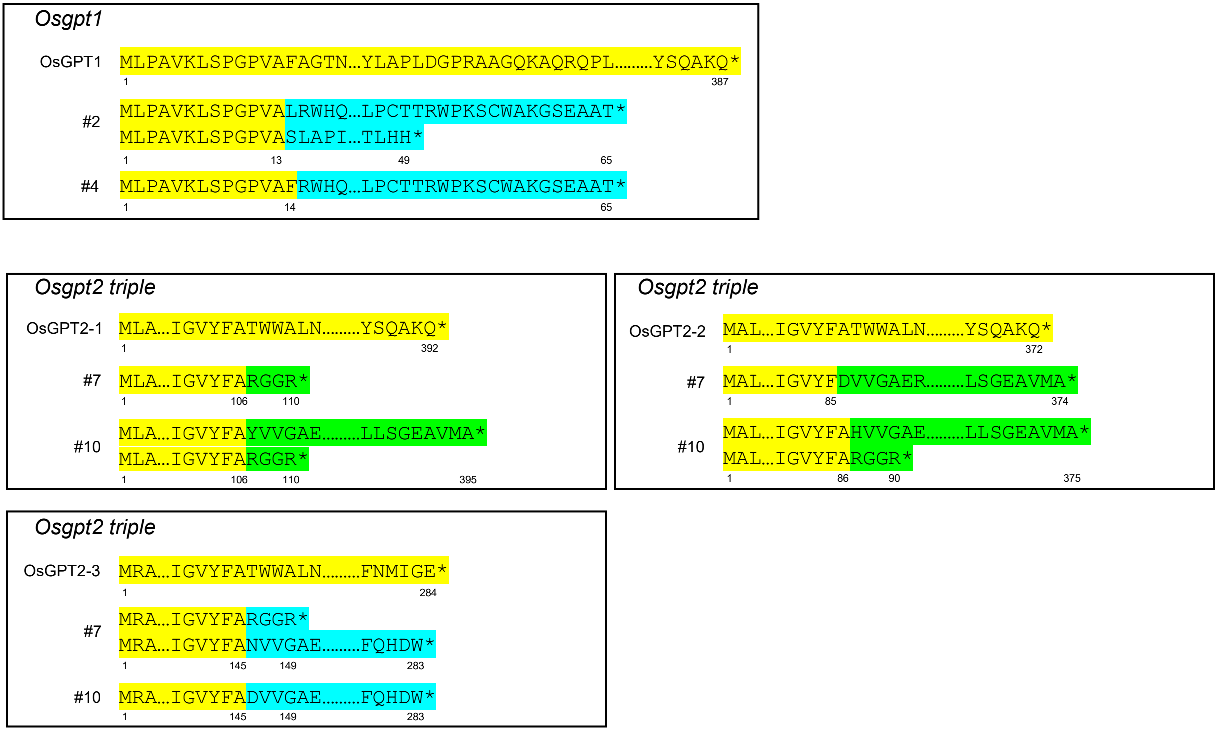

Supplementary Figure S4. Predicted OsGPT protein sequences encoded by CRISPR/Cas9-induced mutant alleles. Predicted amino acid sequences of OsGPT1 and OsGPT2 proteins in *Osgpt1* and *Osgpt2 triple* mutants. Yellow boxes indicate wild-type-derived amino acid sequences, whereas colored boxes indicate mutant-specific amino acid sequences generated after frameshift mutations. Dots indicate omitted amino acid residues, and asterisks indicate stop codons. The numbers correspond to the amino acid positions immediately before and after the mutation-derived sequence and the position of the last amino acid residue in the truncated protein.

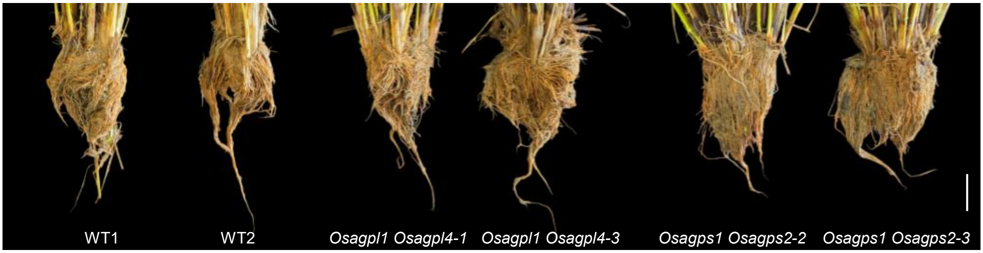

Supplementary Figure S5. Representative root-system images of WT, *Osagpl1 Osagpl4*, and *Osagps1 Osagps2* plants. No obvious gross root morphological differences were observed between WT and the AGPase double-mutant lines under the examined conditions. Scale bar = 5 cm.
